# Is Gestational Diabetes Mellitus Associated with Peripartum Infections?

**DOI:** 10.3390/microorganisms13092030

**Published:** 2025-08-30

**Authors:** Manal Massalha, Rula Iskander, Bibiana Chazan, Enav Yefet, Zohar Nachum

**Affiliations:** 1 Department of Obstetrics and Gynecology, Emek Medical Center, Afula 1834111, Israel; noola87@gmail.com (M.M.); rula_isk@hotmail.com (R.I.); nachum.zo@gmail.com (Z.N.); 2 The Ruth and Bruce Rappaport Faculty of Medicine, Technion-Israel Institute of Technology, Haifa 3200003, Israel; 3 Infectious Diseases Unit, Emek Medical Center, Afula 1834111, Israel; chazan_b@clalit.org.il; 4 Department of Obstetrics and Gynecology, Tzafon Medical Center, Poriya 1528001, Israel; 5 Azrieli Faculty of Medicine, Bar-Ilan University, Safed 1311502, Israel

**Keywords:** endometritis, chorioamnionitis, gestational diabetes mellitus, pregnancy, peripartum infection, postpartum

## Abstract

We investigated the association between gestational diabetes mellitus (GDM) and the rate of peripartum infections (chorioamnionitis and/or endometritis). A retrospective cohort study was conducted using data collected between January 2014 and July 2021. The study group comprised women with GDM, while the control group consisted of women without GDM, matched for age ≥ 35 Y, primiparity, pre-gestational body mass index (BMI), cesarean and vacuum deliveries, and preterm deliveries. The primary outcome was the rate of peripartum infections. Data from 1683 GDM women and 1683 matched controls were analyzed. No significant difference was observed in the rate of peripartum infections between the GDM and control groups (26 (1.5%) versus 14 (0.8%), respectively; *p* = 0.056), nor in the rates of other infections. After controlling for epidural analgesia rate, BMI, age, and delivery week in multivariable logistic regression, the rate of peripartum infections remained statistically insignificant between the GDM group and controls (OR 1.8, 95% CI 0.9–3.4). The main pathogens isolated in cases of peripartum infections were similar in both groups, primarily consisting of *Escherichia coli* and *Group B Streptococcus*. No difference in the rate of study outcomes was observed when vaginal and cesarean deliveries were analyzed separately. Altogether, GDM was not associated with an increased risk for peripartum infections.

## 1. Introduction

Peripartum infections are defined as chorioamnionitis and postpartum endometritis, both of which are prevalent obstetric complications with significant implications for both maternal and neonatal health [1].

Chorioamnionitis, an inflammatory infection that can develop during labor, affects various components of the intrauterine environment, including the amniotic fluid, placenta, fetus, fetal membrane, or decidua [2]. This condition, when manifesting in the postpartum period, is referred to as endometritis, characterized by inflammation of the endometrium and uterine tissues. Epidemiological data indicate that the incidence of chorioamnionitis during labor is approximately 4% and the rate of puerperal endometritis is 2% [3].

The etiology of intrauterine infection is typically polymicrobial, involving both aerobic and anaerobic bacteria that originate from the vaginal flora [4]. It is important to note that the vaginal microbiome is highly susceptible to hormonal fluctuations. Pregnancy induces a state of relative immunosuppression, which, coupled with alterations in the vaginal flora, predisposes women to an increased risk of vaginal fungal and bacterial dysbiosis and infections, such as bacterial vaginosis and vulvovaginal candidiasis. This shift in the microbial environment inherently elevates the risk of obstetric complications, with a particular emphasis on chorioamnionitis [5,6,7,8].

The consequences of intrauterine infection are far-reaching and severe. Neonatal morbidity associated with this complication encompasses a range of acute conditions, including pneumonia, meningitis, sepsis, and in extreme cases, death. Moreover, the long-term sequelae extend to metabolic and psychiatric disorders [2,9,10,11,12]. The maternal risks are equally concerning, with increased incidence of postpartum hemorrhage, endometritis, peritonitis, and potentially fatal sepsis [2].

Gestational diabetes mellitus (GDM) emerges as a significant factor in the context of intrauterine infections. The condition is characterized by poor glycemic control, elevated body mass index (BMI), and impaired leukocyte function [13,14]. These physiological alterations create a milieu conducive to vaginal flora dysbiosis and infections. Several studies have established an association between GDM and disturbances in vaginal flora, as well as an increased incidence of vaginal infections [15,16,17]. A recent investigation delved into the relationship between GDM, abnormal vaginal flora, and obstetric outcomes. The findings revealed that women with GDM exhibited a fourfold higher incidence of chorioamnionitis or perinatal fever compared to their non-GDM counterparts [18].

GDM-related hyperglycemia and chronic metabolic inflammation have been proposed to increase susceptibility to uterine infections by inducing placental immune alterations—such as changes in immune cell infiltration within the villous tree, upregulation of stress- and inflammation-associated genes, and enhanced release of proinflammatory cytokines like IL-1β, IL-6, and IL-8 from trophoblasts. Furthermore, pronounced hyperglycemia has been shown to elevate placental cytokine production, suppress beta defensin synthesis, and weaken innate placental defenses against infections caused by *E. coli* and *Streptococcus agalactiae* [19,20,21,22,23,24,25,26].

To date, no effective preventative measures or treatments specifically tailored for women with GDM to reduce the risk of peripartum infections are in routine use.

Given the compelling evidence suggesting a link between GDM and intrauterine infections, this study aimed to investigate the prevalence of peripartum infections, specifically chorioamnionitis and endometritis, among women with GDM compared to non-GDM pregnant women.

## 2. Methods

### 2.1. Study Design

A retrospective cohort study was conducted at Emek Medical Center, a university teaching medical center in Afula, Israel, utilizing data gathered between January 2014 and July 2021. The protocol was endorsed by the institutional review board (EMC-006-25). Inclusion criteria encompassed women who gave birth and had data regarding GDM screening and diagnosis and whose body temperature was measured during labor and postpartum. GDM was assessed according to the two-step procedure, beginning with the first step, known as the 50 g glucose challenge test (GCT). In this initial phase, a non-fasting woman was administered a 50 g glucose solution, and blood glucose level was measured one hour later. A glucose level equal to or greater than 200 mg/dL was suggestive of GDM. If the measured glucose level was between 140 and 199 mg/dL, the woman proceeded to the second step. The second step involved conducting the 100 g oral glucose tolerance test (OGTT), which was performed in a fasting state. During this phase, the woman consumed a 100 g glucose solution, and blood samples were collected at four time points: fasting, one hour, two hours, and three hours after ingestion of the glucose. The diagnosis of GDM was established if there were at least two abnormal values according to the Carpenter and Coustan criteria [27] or at least one abnormal value using the National Diabetes Data Group criteria [28]. We excluded women with pre-gestational DM and women who did not complete GDM screening and diagnosis.

All participants were subjected to the same intrapartum protocol regarding infection assessment. The protocol was not changed during the study period.

Intrapartum fever was defined as a single oral temperature of ≥39 °C or an oral temperature of 38–38.9 °C that persisted when re-measured after 30 min [2]. Postpartum fever was characterized by a temperature elevation occurring after delivery using the same criteria.

### 2.2. Study Groups

The study population was stratified into two cohorts: the study group, comprising women diagnosed with GDM, and the control group, consisting of pregnant women without GDM. To minimize confounding factors, the control group was matched to the study group in a 1:1 ratio based on several key demographic and obstetric variables. The matching criteria included maternal age, categorized as 35 years or older versus younger than 35 years; parity, classified as primiparous versus multiparous; pre-gestational body mass index (BMI), stratified as 30 kg/m^2^ or greater versus less than 30 kg/m^2^; mode of delivery, distinguished between operative deliveries (cesarean delivery and vacuum-assisted) and spontaneous vaginal delivery; and gestational age at delivery, defined as preterm (less than 37 weeks of gestation) versus term (37 weeks of gestation or greater).

### 2.3. Outcomes

The primary outcome was the incidence of peripartum infection, specifically chorioamnionitis and/or endometritis. Chorioamnionitis was defined by the American college of obstetricians and gynecologists (ACOG) as the combination of maternal intrapartum fever along with one or more of the following: maternal leukocytosis, purulent cervical discharge, or fetal tachycardia [2].

Endometritis was defined by the centers for disease control and prevention (CDC) as the presence of at least one of the following criteria: (1) the identification of organism(s) from endometrial fluid or tissue through culture or non-culture-based microbiological testing methods, conducted for clinical diagnosis or treatment purposes; or (2) suspected endometritis accompanied by at least two of the following signs or symptoms: fever (>38.0 °C), uterine or abdominal pain or tenderness without another identified cause, or purulent uterine drainage [29].

Secondary outcomes were rate of fever, other infections such as urinary tract infections, mastitis, and surgical site infections. Finally, data regarding the responsible pathogens were also collected.

### 2.4. Data Collection

Comprehensive data were extracted from women’s clinical records, encompassing a broad spectrum of maternal and neonatal parameters. Maternal information included demographic characteristics such as age, anthropometric measurements (weight and height), comprehensive medical history detailing chronic illnesses, smoking status, GDM screening results, GDM treatment (diet alone or hypoglycemic medications including metformin, glibenclamide, and/or insulin), and hemoglobin A1C (HbA1C) level.

Metabolic screening parameters comprised glucose challenge test (GCT) and oral glucose tolerance test (OGTT) outcomes, including the precise gestational age at test administration. Additional maternal data incorporated antepartum complications, with particular emphasis on conditions like hypertension.

In cases of suspected uterine infection, a detailed infectious disease profile was documented. This included antibiotic administration specifics at birth, encompassing antibiotic type, treatment initiation time, and duration. Microbiological investigations were comprehensively recorded, including cultures from products of conception, pregnancy, and peripartum urine cultures and blood cultures as well as diagnoses of associated conditions such as mastitis or phlebitis.

Obstetrical information captured mode of delivery, intrapartum fever occurrence and, when infection was suspected, additional maternal vital signs and detailed fetal heart rate characteristics.

Neonatal outcome assessment included Apgar scores at 1 and 5 min, cord arterial pH, birthweight, and gender. In instances of suspected intrauterine infection, specific neonatal infectious signs were meticulously documented.

Socioeconomic status was assessed using the classification system established by the Israeli Central Bureau of Statistics, which assigns each municipal authority a score from 1 to 10. The ranking is based on a composite index incorporating residents’ income sources (e.g., employment, social benefits), housing conditions (including crowding and quality), ownership of household appliances, vehicle availability, educational attainment, employment characteristics, and additional demographic indicators [30]. For the purposes of this study, municipal authorities ranked in clusters 7 through 10 were defined as high socioeconomic status. This threshold aligns with commonly accepted divisions in Israeli population studies [31].

### 2.5. Statistical Analysis

The rate of peripartum infections in the general population is around 6% [3]. Assuming that the incidence of peripartum infection in women with GDM would be higher, reaching 9% compared with 6% among women without GDM, a total of 3236 women were needed for the study (5% 2-sided alpha, 90% power).

Intercohort baseline characteristics and outcomes were compared using the Student *t*-test (or the Wilcoxon two-sample test) for continuous variables and χ^2^ (or Fisher’s exact test) for categorical variables. Adjustment for confounding variables was performed utilizing multivariable logistic regression analysis. A *p* value > 0.05 in the Hosmer and Lemeshow Goodness-of-Fit test represented an appropriate model fit. The results are presented as odds ratios (ORs) with 95% confidence intervals (CIs). Collinearity diagnostics were performed using eigenvalues and the Condition Index. Sub-analyses were performed for vaginal and caesarean deliveries. Additional sub-analyses for the rate of peripartum infections were conducted according to GDM treatment and up to and above the mean HbA1C of the GDM population in this study (5.2 ± 0.5%). Statistical analyses were carried out with SAS version 9.4 (SAS Institute, Cary, NC, USA). Significance was set at a *p* value < 0.05.

## 3. Results

Overall, 1683 women had GDM and met the inclusion criteria, with 1683 matched controls without GDM.

The obstetrics and demographic variables of both groups are presented in Table 1. A statistical difference was found in variables maternal age, pre-gestational BMI, and the rate of epidural analgesia, which were higher in the GDM group, while delivery week was lower in the GDM group compared to the controls.

Maternal outcomes are presented in Table 2. There was no difference in the rate of peripartum infections between the GDM and control groups (26 (1.5%) vs. 14 (0.8%), respectively; *p* = 0.056) nor in the rate of chorioamnionitis, endometritis, or other infections (Table 2). In addition, the rate of peripartum infections was not different between the GDM group and controls after controlling for epidural analgesia rate, BMI, age, hypertensive diseases in pregnancy, and delivery week in multivariable logistics regression (OR 1.7, 95% CI 0.9–3.4, Hosmer and Lemeshow Goodness-of-Fit test *p* = 0.84). The highest Condition Index observed was below two, indicating no evidence of multicollinearity among the independent variables. The rate of all types of infections in univariate analysis was higher in the GDM group compared to controls (41 (2.4%) vs. 24 (1.4%), respectively; *p* = 0.03) but became statistically insignificant in multivariable analysis (OR 1.60, 95% CI 0.95–2.69, Hosmer and Lemeshow Goodness-of-Fit test *p* = 0.73). The highest Condition Index observed was below two as well. The rate of peripartum infections was not different between GDM women treated by diet alone versus women treated with hypoglycemic medications (63/1469 (1.6%) vs. 3/214 (1.4%), respectively; *p* = 1) nor in GDM women with an HbA1C level up to 5.2% (the mean HbA1C of the GDM population) or above (10/573 (1.7%) vs. 6/397 (1.5%), respectively; *p* = 0.78). In order to evaluate a possible trend in the overall rate of peripartum infections, we compared the rate of peripartum infections during 2014–2017 vs. 2018–2021. There was no difference between the groups (20 (1%) vs. 20 (1.5%), respectively; *p* = 0.23).

The types of isolated microorganisms are presented in Table 3, without apparent difference between the groups.

There was no difference in the rate of the study outcomes when vaginal deliveries (Table 4) and CDs (Table 5) were analyzed separately.

## 4. Discussion

In the present study, we evaluated the association between GDM and peripartum infections. GDM was not associated with peripartum infections or any other infection during delivery and the postpartum periods. It is noteworthy that the general prevalence of peripartum infections was lower than the mean rate reported in a previous meta-analysis, though the range of peripartum infections varied between 0.6–19.7% for chorioamnionitis and 0–16.2% for endometritis in different studies [3].

The association between GDM and infections has been a subject of considerable interest in previous studies. A recent meta-analysis that included 16 studies analyzed the rate of infections among 111,649 GDM women vs. 1,429,659 non-GDM women [17]. A significant association between GDM and infections was demonstrated (pooled-OR 1.3, 95% CI [1.2–1.5]). Sub-analyses showed a significant association for bacterial infections (pooled-OR 1.2, 95% CI [1.1–1.4]), UTI (pooled-OR, 1.2 95% CI [1.1–1.3]) and SARS-CoV-2 (pooled-OR 1.5, 95% CI [1.2–2.0]) but not for vaginal candidiasis or gingivitis. The results underscore the significance of acknowledging GDM as a risk factor for infections [17].

Nevertheless, while the association between glycemic control and infections is well established in type 1 and type 2 diabetes unrelated to pregnancy [32,33,34], it has not been specifically studied in GDM, and further research is needed in this area. In addition, larger prospective cohorts are needed to evaluate the association between GDM and specific types of peripartum infections.

Hyperglycemia is known to cause immune dysfunction, adversely affecting neutrophil chemotaxis, macrophage function, and phagocytic responses, leaving diabetic patients more susceptible to infections and related comorbidities [35]. GDM was also shown to induce immune system dysfunction, and the pathophysiological mechanisms underlying this process are elucidated by the deleterious effects of diabetes on critical immune processes, similar to patients with type 2 diabetes mellitus. Maternal hyperglycemia ensues, instigating a ‘glucose stress’ response and concurrent systemic inflammation. Previous findings have proposed that both placental and visceral adipose tissue play a part in instigating and mediating this low-grade inflammatory response, which involves altered infiltration, differentiation, and activation of maternal innate and adaptive immune cells. The resulting maternal immune dysregulation is responsible for exacerbation of the condition and a further reduction in maternal insulin sensitivity [36]. Consequently, this compromised immune function may result in increased infection susceptibility among GDM women. Furthermore, suboptimal glycemic control may alter vaginal microbiome [37]. A healthy vaginal microbiome plays a key function in the prevention of bacterial vaginosis, candidiasis, and various ascending bacterial infections [38,39]. Lactobacillus constitutes seventy percent of the vaginal flora [18,40], and it is believed to play a defensive role in the vaginal immune system through the production of an acidic environment, which in turn has a harmful impact on pathogens invading the tract [39]. GDM was also shown to cause a shift in the colonization of the lactobacilli from lactobacilli crispatus towards lactobacilli acidophilus [18]. The meaning of this alteration is yet to be determined.

While previous investigations have offered insights into the associations between GDM and various infections during pregnancy [17], there is a paucity of research specifically examining the relationship between GDM, chorioamnionitis, and endometritis. Only one small-scale study investigated the outcome of GDM among Bangladeshi urban women and found the rate of puerperal sepsis to be 14/72 (19%) vs. 3/72 (4%) of women with and without GDM, respectively; *p* < 0.05 [41]. These results are different than the results of the current study, which did not demonstrate increased risk for peripartum infections in women with GDM. A plausible explanation for our findings may be attributed to the utilization of more contemporary data in our study, during a period when stringent glycemic control was more strongly recommended and implemented in clinical practice. This temporal context reflects evolving guidelines and increased awareness of the importance of tight glucose management in GDM, potentially mitigating some of the associated risks observed in the earlier study. Future studies should be performed, especially in high-income countries, in order to evaluate this hypothesis.

As demonstrated, the existing literature lacks comprehensive analyses of the association between GDM and peripartum infections, necessitating further investigation to elucidate this relationship more precisely. The present study addresses this significant gap in the literature by utilizing data derived from a large cohort and accounting for potential confounding variables. This approach allows for a more robust examination of the associations between GDM, chorioamnionitis, and endometritis, providing valuable epidemiological evidence to inform clinical practice and guide future research in this domain.

The strengths of this study are the use of a large electronic database in which the data were documented in real time with a minimal rate of missing data and the use of a matched control group to address various possible confounders for the same period of time. The limitations of this study are its retrospective design and the limited data regarding glycemic control, as daily glucose charts were unavailable. Future prospective studies with large cohorts should focus on the association between glycemic control and the risk of various types of peripartum infections. Moreover, both groups demonstrated significantly reduced rates of peripartum infection than what was assumed. This significant overestimation compromises power: the observed absolute difference of 0.7% may indicate a type II error rather than genuine equality; in order to demonstrate a significant difference of 0.7% between the groups, a total sample size of 7282 women would be required (5% two-sided alpha, 80% power).

## 5. Conclusions

In this study, we did not find an association between GDM and increased risk for peripartum infections.

## Figures and Tables

**Table 1:** Demographic and obstetric variables.

Variable	GDM Group (*n* = 1683)	Control Group (*n* = 1683)	*p*-Value
Age (years)	31.7 ± 5.6	30.9 ± 6.9	<0.0001
Age ≥ 35 *	546 (32%)	546 (32%)	1
High socioeconomic status €	22 (1.3%)	35 (2.1%)	0.08
Smoking	139 (8%)	113 (7%)	0.09
Delivery week	38.2 ± 1.6	38.7 ± 2.0	<0.0001
Birth weight (gr)	3198 ± 529	3211 ± 547	0.68
Primiparity *	572 (34%)	572 (34%)	1
Pregnancy number	3.1 ± 2.1	3.0 ± 2.0	0.12
Birth number	2.5 ± 1.6	2.5 ± 1.5	0.33
Pre-gestational BMI (kg/m^2^)	27.4 ± 5.8	26.0 ± 6.0	<0.0001
Pre-gestational BMI ≥ 30 kg/m^2^ *	476 (28%)	476 (28%)	1
Hypertensive disease in pregnancy **	179 (11%)	129 (8%)	0.003
Autoimmune disease	14 (0.8%)	21 (1.3%)	0.24
Prophylactic antibiotic around delivery	448 (27%)	426 (25%)	0.39
Epidural	466 (28%)	414 (25%)	0.04
Preterm delivery *	145 (9%)	145 (9%)	1
Cesarean delivery *	453 (27%)	453 (27%)	1
Emergent cesarean delivery	206 (12%)	227 (13%)	0.28
Operative delivery *	64 (4%)	69 (4%)	0.66
Manual exploration of uterine cavity	64 (3.8%)	71 (4.2%)	0.54
Neonate gender—Males	888 (53%)	886 (53%)	0.76
Peripartum infection in previous delivery ***	71/894 (8%)	53/862 (6%)	0.14

Data are mean ± standard deviation or number (%). * Variable represents the rate in the GDM group. The rate in the control group was matched. ** Includes chronic hypertension, gestational hypertension, and preeclampsia. *** Data were missing when past delivery was elsewhere. € Thirty-six women lived in places of residence that were not ranked due to their small population size.

**Table 2:** Maternal outcomes.

Outcome	GDM Group (*n* = 1683)	Control Group (*n* = 1683)	*p*-Value
All types of infections	41 (2.4%)	24 (1.4%)	0.03
Peripartum infection	26 (1.5%)	14 (0.8%)	0.056
Chorioamnionitis	22 (1.3%)	13 (0.8%)	0.13
Endometritis	22 (1.3%)	14 (0.8%)	0.18
Urinary tract infection	11 (0.7%)	7 (0.4%)	0.34
Mastitis	0	0	-
Respiratory infection	0	0	-
Phlebitis	0	0	-
Positive cultures *	18 (1.1%)	11 (0.7%)	0.19

* In some patients, more than one culture was positive.

**Table 3:** Type of microorganism from positive cultures.

	Type of Pathogen	
Type of Infection	GDM Group Total Positive Cultures (*n* = 18)	Control Group Total Positive Cultures (*n* = 11)
Chorioamnionitis	*Escherichia* (*E*.) *coli*—5	*E. coli*—2
	*Enterococcus faecalis*—2	*GBS*
	*Group B Streptococcus* (*GBS*)	*Morganella morganii*
	*Gardnerella vaginalis*	*Klebsiella pneumonia*
	*Haemophilus haemolyticus*	
Endometritis	*E. coli*—4	*E. coli*—2
	*GBS*	*GBS*
	*Haemophilus haemolyticus*	*Morganella morganii*
		*Klebsiella pneumonia*
Urinary tract infection	*E. coli*—6	*E. coli*—3
	*Klebsiella pneumonia*—2	*Klebsiella pneumonia*
	*Enterococcus faecalis*—2	*Enterococcus faecalis*—3
	*Enterobacter aerogenes*	*Enterobacter aerogenes*
		*GBS*
Bacteremia	*Haemophilus haemolyticus*	*E. coli*

If several microorganisms and/or several tissues were involved, they are listed in all the relevant rows.

**Table 4:** Maternal outcomes in vaginal deliveries.

Outcome	GDM Group (*n* = 1230)	Control Group (*n* = 1230)	*p*-Value
All types of infections	29 (2.4%)	16 (1.3%)	0.051
Peripartum infection	18 (1.5%)	10 (0.8%)	0.13
Chorioamnionitis	14 (1.1%)	10 (0.8%)	0.41
Endometritis	16 (1.3%)	10 (0.8%)	0.24
Urinary tract infection	8 (0.7%)	5 (0.4%)	0.4
Mastitis	0	0	-
Respiratory infection	0	0	-
Phlebitis	0	0	-
Positive cultures *	13 (1.1%)	8 (0.7%)	0.27

* In some patients, more than one culture was positive.

**Table 5:** Maternal outcomes in cesarean deliveries.

Outcome	GDM Group (*n* = 453)	Control Group (*n* = 453)	*p*-Value
All types of infections	12 (2.7%)	8 (1.8%)	0.37
Peripartum infection	8 (1.8%)	4 (0.9%)	0.25
Chorioamnionitis	8 (1.8%)	3 (0.7%)	0.13
Endometritis	6 (1.3%)	4 (0.9%)	0.52
Urinary tract infection	3 (0.7%)	2 (0.4%)	1
Mastitis	0	0	-
Respiratory infection	0	0	-
Phlebitis	0	0	-
Positive cultures *	5 (1.1%)	3 (0.7%)	0.73

* In some patients, more than one culture was positive.

## Data Availability

The data generated for this manuscript are available from Z.N. following a reasonable request and the approval of the IRB board.
